# Crosstalk between Neuron and Glial Cells in Oxidative Injury and Neuroprotection

**DOI:** 10.3390/ijms222413315

**Published:** 2021-12-10

**Authors:** Kyung Hee Lee, Myeounghoon Cha, Bae Hwan Lee

**Affiliations:** 1 Department of Dental Hygiene, College of Bio-Health Convergence, Dongseo University, Busan 47011, Korea; kyhee@dongseo.ac.kr; 2 Department of Physiology, College of Medicine, Yonsei University, Seoul 03722, Korea; mhcha@yuhs.ac; 3 Brain Korea 21 PLUS Project for Medical Science, College of Medicine, Yonsei University, Seoul 03722, Korea

**Keywords:** neuron–glia interaction, astrocyte, microglia, oxidative injury, neuroprotection

## Abstract

To counteract oxidative stress and associated brain diseases, antioxidant systems rescue neuronal cells from oxidative stress by neutralizing reactive oxygen species and preserving gene regulation. It is necessary to understand the communication and interactions between brain cells, including neurons, astrocytes and microglia, to understand oxidative stress and antioxidant mechanisms. Here, the role of glia in the protection of neurons against oxidative injury and glia–neuron crosstalk to maintain antioxidant defense mechanisms and brain protection are reviewed. The first part of this review focuses on the role of glia in the morphological and physiological changes required for brain homeostasis under oxidative stress and antioxidant defense mechanisms. The second part focuses on the essential crosstalk between neurons and glia for redox balance in the brain for protection against oxidative stress.

## 1. Introduction

The brain is highly susceptible to oxidative injury because of its high rate of oxidative metabolic activity, intense production of reactive oxygen metabolites, weak antioxidant capacity, relatively high lipid content, high energy requirements, non-replicating neuronal cells, and high membrane surface to cytoplasm ratio. Consequently, oxidative injury induces neurodegenerative disease [1,2]. Specifically, reactive oxygen species (ROS) increase vulnerability to brain cell damage and functional decline via a redox imbalance between pro-oxidant and antioxidant agents, which induce the formation of free radicals and other reactive molecules. There have been several studies on neuroprotection and the rescue of neurons after oxidative injury [3,4].

To develop new therapeutic interventions and diagnose the diseases that result from oxidative brain injury, it is necessary to understand the physiological functions of brain cells and the crosstalk between them. Astrocytes are important for brain homeostasis because they provide nutrition to neurons, maintain the integrity of the blood–brain barrier, regulate synapse activity, and process cell metabolites [5]. Microglia are crucial because they function as macrophages in the brain and rapidly respond to disturbances in the brain [6]. Targeting the interaction between astrocytes, microglia, and other brain cells may arrest or reverse oxidative injury, which results in neuroprotection. Specific glial cell-based diagnostic approaches that detect glial cell signaling pathways using biomarkers or neuroimaging may identify individuals at risk of neuronal dysfunction much earlier and more precisely, and these biomarkers may allow for the monitoring of oxidative disease progression and/or recovery. This review summarizes the current knowledge of the physiological roles and functions of astrocytes and microglia in response to oxidative stress, their interactions with neurons, and their neuroprotective capabilities.

## 2. Vulnerability of the Brain to Oxidative Stress

Oxidative stress, which can be induced by various mechanisms, plays a crucial role in neuronal death and brain dysfunction and induces neurodegenerative diseases, including Alzheimer’s disease (AD), Parkinson’s disease (PD), aging, and other neurodegenerative diseases [7,8,9]. The brain requires large amounts of adenosine triphosphate (ATP) and consumes over 25% of the circulating glucose and 20% of total basal oxygen (O_2_) to maintain neuronal activity [10,11]. However, glucose consumption may induce oxidative stress by inactivating proteins through the formation of advanced glycation end products, and oxygen utilization can produce ROS and reactive nitrogen species (RNS) via endogenous mechanisms during cellular respiration [12,13]. ROS/RNS production largely occurs during oxidative phosphorylation, and increased free radical production plays a crucial role in neuronal death. When ROS/RNS production exceeds the scavenging capacity of the antioxidant response system, extensive protein degradation, lipid oxidation, and DNA degeneration occur and subsequently induce an excessive and pathological loss of neurons [14,15].

**Figure 1:**
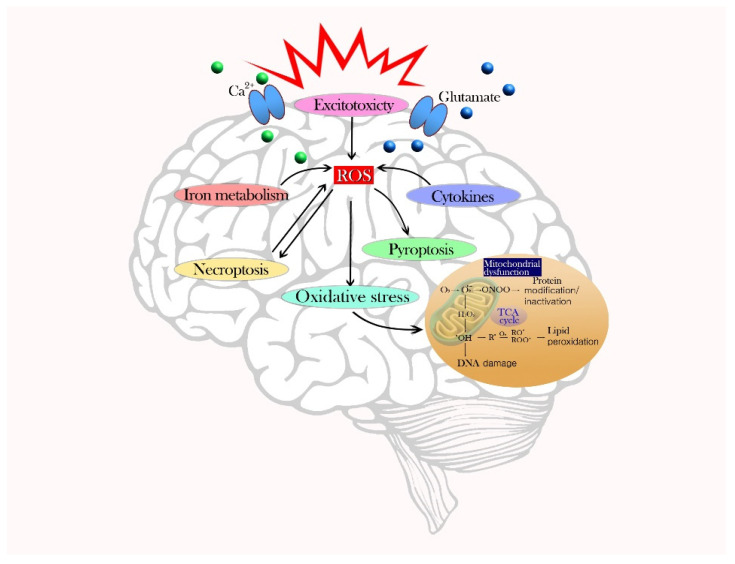
Schematic of the main pathophysiological mechanism of cell death due to oxidative stress. The presence of ROS due to an imbalance of pro-oxidants and antioxidants can damage a variety of cells in the brain. Formation of ROS and mitochondria dysfunction occurs during the primary mechanism of oxidative stress. Secondary mechanisms of cell death by ROS production include excitotoxicity, iron metabolism, cytokines, pyroptosis, and necroptosis, which amplify cell death.

The primary mechanism of oxidative cell death is the formation of ROS and mitochondria dysfunction. Single-electron reactions produce reactive molecules as undesirable side-products of respiration or as a result of excess defense mechanisms. ROS/RNS include singlet oxygen, superoxide anion radicals, hydroxyl radicals, hydrogen peroxide, nitric oxide, and peroxynitrite anions [16]. These unstable molecules destroy cellular lipids and proteins, and consequently activate intracellular ROS production via nicotinamide adenine dinucleotide phosphate (NADPH) and the electron transport chain. Through the cell membrane, NADPH is used as an electron donor for electron transfers and, ultimately, molecular oxygen is reduced to ROS [17]. The mitochondria are the main sites of intracellular ROS production and the targets of ROS-induced injury. Slow electron transfer during the respiratory chain increases ROS production and seriously damages the antioxidant system [18]. Secondary mechanisms of cell death via ROS production are excitotoxicity, iron metabolism, cytokines, pyroptosis, and necroptosis. The excessive release of glutamate and an influx of Ca^2+^ causes calcium overload in neurons and a disturbance in intracellular Ca^2+^ homeostasis, which can intensify excitotoxicity by leading to ROS production [19]. Iron-dependent oxidative stress also causes brain function deterioration. When an overload of iron overwhelms a cell’s detoxification systems, iron content (especially Fe^2+^) increases and promotes the conversion of H_2_O_2_ to ^•^OH through the Fenton reaction, thereby amplifying oxidative stress [20]. Inflammatory cells, immune factors, and chemokines can release harmful compounds and cytokines that exacerbate oxidative stress and impair neurons. Microglia, which are important for redox stability, activate NADPH oxidase (NOX) and nitric oxide synthase (NOS) enzymes, leading to an increased production of ROS and RNS [21,22]. Astrocytes stimulate the activation and proliferation of microglia, which produce many inflammatory mediators in the brain. Pyroptosis is another type of inflammatory programmed cell death. The leucine-rich-repeat (NLR) pyrin-domain-containing 3 (NLRP3) inflammasome signaling pathway that induces cell pyrolysis is triggered by ROS generation during brain injury [23]. NLRP3 inflammasome activation in astrocytes and microglia induce inflammatory responses and neuronal death [24]. Intracellular ROS accumulation can alter proteins, glucose, lipids, and nucleic acids to cause cell dysfunction and death. Tumor necrosis factor (TNF)-induced necroptosis (programmed necrosis) can also lead to ROS generation [25]. The pathophysiological mechanism of cell death due to oxidative stress is described in Figure 1.

## 3. Astrocytes

### 3.1. Astrocytes in the Brain

Astrocytes are the most dynamic and abundant cells in the human brain and are responsible for maintaining brain homeostasis. Astrocytes are called territorial cells and have several extended processes that communicate with adjacent cells; thus, they form organized anatomical domains with associated functional syncytia [26]. Astrocytes project vascular processes (astrocytic end-feet) onto intraparenchymal blood vessels and ensheath the vessel surfaces to control the movement of molecules and cells between the vascular compartment and the brain [27]. Human astrocytes are usually classified into four subdivisions based on their neuroanatomy [28]. First, interlaminar astrocytes have a round cell body and short processes and are located in layer I of the cortex. Second, protoplasmic astrocytes are found in gray matter and are located in layers II–VI of the cortex. They are the most abundant astrocytes and have numerous processes and a bushy morphology. Third, varicose projection astrocytes are located in layers V–VI and have short spiny processes with from one to five longer processes that may function in long-distance communication within the cortex. Fourth, fibrous astrocytes are located in white matter and are larger cells containing fewer processes. Fibrous astrocyte processes send numerous extensions to contact oligodendroglia that wrap myelinated axons [29]. Astrocytes are also classified into type I–III according to their morphological characteristics, such as cell body size, number of processes, thickness of processes, direction of processes, and length of processes. Type I astrocytes are characterized by a small cell body and numerous short processes. Type II astrocytes are characterized by a bipolar shape and long processes. Type III astrocytes are characterized by a star shape and long processes [30,31]. The function of astrocytes is to aid neurons by playing supportive roles in synaptic function and the modulation of neurotransmission. The processes of astrocytes ensheath synapses and contain a variety of receptors for neurotransmitters, cytokines, growth factors, and ion channels. Astrocytes are affected by intracellular Ca^2+^ release by extracellular glutamate, and maintain the ionic balance of synapses by increasing intracellular Ca^2+^ levels following the secretion of numerous gliotransmitters, such as glutamate, purines, GABA, and D-serine [32,33] Neurons are highly sensitive to small changes in the brain microenvironment, even though their metabolic consumption is high. The role of astrocytes in the normal brain is the maintenance of extracellular homeostasis through glutamate uptake and recycling, K^+^ buffering, supplying energy substrates, pH buffering, and defense against oxidative stress [28].

### 3.2. Astrocytes in Oxidative Injury

**Figure 2:**
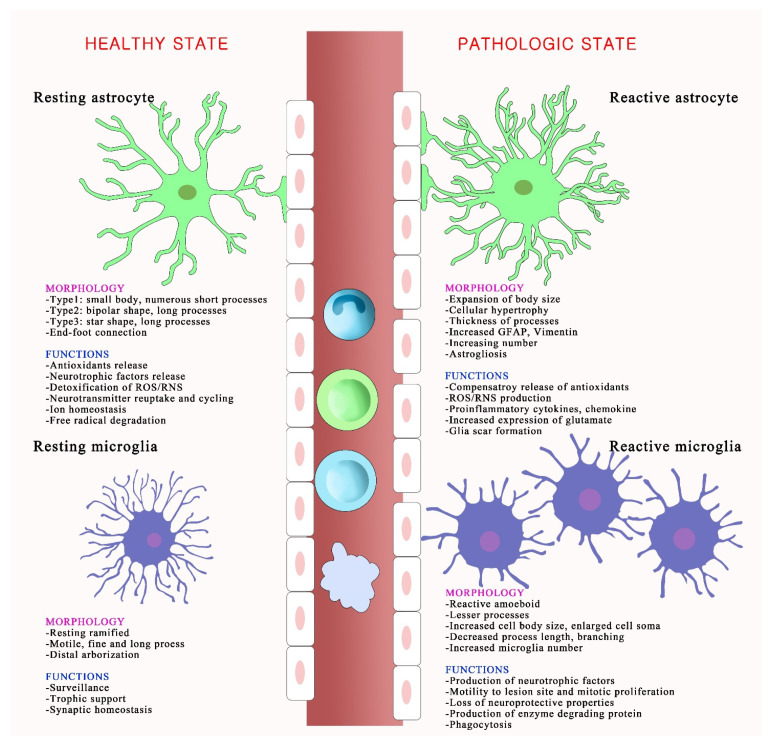
Schematic depicting morphological and functional changes in activated glial cells following oxidative stress. The resting and reactive states of astrocytes and microglia have different morphologies and functions. In a healthy brain, astrocytes are called territorial cells and they maintain extracellular homeostasis via numerous cellular processes. Microglia use their defense mechanisms to rapidly respond to disturbances in the brain environment, and assist in specific immune functions. However, under oxidative stress, reactive astrocytes undergo astrogliosis, which associates with cellular hypertrophy, astrocyte proliferation, increasing numbers and thickness of processes, and expanded cell body size. Reactive microglia, also called amoeboid microglia, exhibit morphological modifications and proliferation and produce several inflammatory mediators, including nitric oxide and superoxide. The disruption of vascular integrity is observed and increases the permeability of immune cells in pathological conditions.

Astrocytes exist in a resting or reactive state in the brain, as shown in Figure 2. Reactive astrocytes release inflammatory cytokines including TNF and ROS, and form glial scars that impede axon regeneration and neurite outgrowth [34,35,36]. Activated astrocytes aid in the recovery of brain function after injury but can be neurotoxic. Reactive astrocytes release nitric oxide (NO) into the extracellular space; this can lead to neuronal injury and death by increasing lipid peroxidation, mitochondrial impairment, and inducing DNA strand breaks [37]. The astrocytic antioxidant system balances ROS (superoxides, hydroxyl radicals, and nitric monoxide) that are naturally produced during oxygen metabolism by the CNS [38]. Oxidative stress in reactive astrocytes leads to long-term effects on specific proteins, including connexins, glutamate transporters, and enzymes, which affect interactions between astrocytes and neurons [39]. The glutamate uptake by an astrocyte requires a high level of energy, needing more than one ATP molecule for one glutamate take-up. However, the lack of ATP is related to the mechanisms of ROS-induced glutamate uptake blockade in astrocytes [40,41]. Blocking astrocyte glutamate transporters increases neurotoxicity by potentiating neuronal excitability and excitatory neurotransmission [42]. Oxidative stress generated by astrocytes mainly occurs through mitochondria-derived oxidative stress, NADPH-derived oxidative stress, and RNS production. Mitochondria are distributed in the cell body and in the thin and long processes of astrocytes [43]. Disrupting mitochondrial function and increasing ROS in astrocytes lead to astrogliosis. NADPH-derived oxidative stress significantly affects the physiological function of astrocytes. Among the NOX family, NOX2 and NOX4 are the most abundantly expressed NOX isoforms in the CNS [43]. NOX4, but not NOX2, is expressed in astrocytes, and even a low expression of NOX4 regulates oxidative stress in astrocytes [44,45]. Astrocytic RNS production also affects astrocyte-derived oxidative stress. The main NOS isoforms, including Ca^2+^/calmodulin-dependent neuronal NOS, endothelial NOS, and Ca^2+^-independent inducible NOS, are observed in astrocytes [5,46]. Astrocytic NO leads to astrocyte-induced neuronal degeneration and Cu-Zn superoxide dismutase (SOD1) aggregation in astrocytes, which may induce ischemic/reperfusion CNS injury [47,48].

### 3.3. Astrocyte-Medicated Antioxidant Defense

Astrocytes are the main cells that maintain glutamate homeostasis, which indirectly affects the balance of oxidative stress, by regulating excitatory amino acids. Astrocytes also prevent excitotoxicity by releasing neurotrophic factors, such as glial-cell-line-derived neurotrophic factor (GDNF) and nerve growth factor (NGF), which support neuronal survival [39,49]. For neuroprotection during oxidative stress, astrocytes produce a variety of antioxidant molecules, including GSH, ascorbate, and vitamin E, and activate ROS-detoxifying enzymes, such as GSH S-transferase, GSH peroxidase, thioredoxin reductase, and catalase to improve neuronal survival [26,50,51]. Moreover, astrocytes participate in metal sequestration in the brain to prevent the generation of free radicals by redox-active metals. Astrocytes express high levels of metallothioneins and ceruloplasmin, which are involved in metal binding and ion trafficking [52].

Astrocytes can synthesize the GSH tripeptide with glutamate cysteine ligase and GSH synthetase. Astrocytes release GSH into the extracellular space and neurons take up the GSH directly or use extracellular neuronal aminopeptidase N to form glycine and cysteine [53]. A reduced neuronal protection against oxidative injury was observed in GSH-depleted astrocytes by limiting the substrate for GSH synthesis in neurons [54]. Astrocytes increase the capacity to synthesize GSH by increasing the capacity to uptake cysteine, thereby enhancing the neuroprotective effect of astrocytes against oxidative stress [5]. Another astrocyte antioxidant defense mechanism is the recycling of ascorbate, which can directly scavenge ROS and act as a cofactor for the recycling of oxidized vitamin E and GSH [2]. This recycled ascorbate is used intracellularly in astrocytes and/or released into the extracellular space for neurons to use for their own antioxidant defense mechanism. When ascorbic acid enters neurons, it inhibits glucose consumption and stimulates lactate transport. Ascorbic acid regulates the astrocyte-neuron lactate shuttle [55], and neurons produce glutamate, which stimulates ascorbic acid release from astrocytes during glutamatergic synaptic activity [56,57]. In the Nrf2-Keap1-ARE pathway, an important endogenous antioxidant system in the CNS, the ROS-inducible transcription factor nuclear factor erythroid 2-related factor 2 (Nrf2), regulates the GSH system, the thioredoxin system, and SOD [58]. Nrf2 is produced and ubiquitinated for degradation by binding to the Kelch-like ECH-associated protein 1 (Keap1) under basal conditions [59]. However, Keap1 binding to Nrf2 is inhibited by increased oxidative stress conditions, and this allows Nrf2 to escape degradation and interact with antioxidant response elements (AREs) in gene promoters [60,61]. Astrocytes show higher basal and stimulated levels of ARE binding by Nrf2 than neurons [62]. In addition, tertiary butylhydroquinone (tBHQ) activates Nrf2 and its downstream antioxidant enzymes, such as reduced coenzyme/quinone oxidoreductase 1 (NQO1), in astrocytes, but not in neurons [63]. Astrocytic Nrf2 is the main regulator of oxidative homeostasis as determined by the observation that Nrf2−/− astrocytes have more severe inflammatory responses. Further, astrocytic dopamine D2 receptor regulates GSH synthesis via Nrf2 transactivation in vivo [64,65].

## 4. Microglia

### 4.1. Microglia in the Brain

Microglia, which have numerous fine and motile processes that survey the parenchymal environment, represent approximately 10% of CNS cells. Each microglial cell has its own territory, which is approximately 50 µm in diameter [66]. Microglia, referred to as the resident macrophages in the CNS, are long-lived and self-renewing cells. In a healthy brain, microglia have a ramified morphology and are in a “quiescent” or “resting” state [67]. Microglial processes undergo continuous cycles of extension and withdrawal, scan their environment for disruptions in brain homeostasis, and systematically synapse to monitor and regulate neuronal activity via a specific signaling mechanism [68,69]. Microglia change their morphology from the resting state to the reactive amoeboid state during a pathological brain condition. Reactive microglia, which evolve into phagocytic or amoeboid microglia, have an increased cell body size, fewer processes, reduced process length and branching, and increased numbers and proliferation, indicating an intimate link between morphology and function [70,71,72,73] (Figure 2). Microglia are highly sensitive to environmental signals and respond to maintain their homeostatic phenotype in a disease-specific and brain-region-specific manner. White and gray matter microglia show a different immune regulation; cortex-associated microglia play a role in neurodegeneration and white-matter-associated microglia play a role in de-/remyelination [74].

Usually, activation of the neurotransmitter receptors inhibits the inflammatory activation of microglia and inhibits the production of abnormal molecules and abnormal concentrations of physiological molecules. Once activated upon brain injury or infection, microglia initiate immune responses and produce a number of cytokines, chemokines, and growth factors, and upregulate the expression of cell surface receptors, such as toll-like receptors (TLRs), phagocytic receptors, scavenger receptors, and various complement factors [75,76]. Microglia express several neurotransmitter receptors, including GABA, glutamate, dopamine, and noradrenaline [66,77].

### 4.2. Microglia in Oxidative Injury

During oxidative stress, activated microglia produce several inflammatory mediators, including NO and superoxide, which freely cross the cell membrane and act as signaling molecules. NO and superoxide can form peroxynitrite, which causes DNA fragmentation, lipid oxidation, and induces neuronal death [78,79]. In cultured microglia, superoxide production, which is catalyzed by nitrates/nitrites (NO_x_), is induced by phorbol ester, and NO production is stimulated by the induction of iNOS upon treatment with bacterial lipopolysaccharide (LPS) and interferon-γ (IFNγ) [80,81]. The expression of iNOS after intrahippocampal treatment with LPS was induced more rapidly in microglia than in astrocytes, and a lower concentration of LPS was required for iNOS induction in microglia than in astrocytes [82,83]. In addition, arginine is a well-known physiological substrate of NOS. Activated microglia with an insufficient amount of arginine leads to iNOS-mediated production of NO and superoxide, which form toxic peroxynitrite [84]. The induction of iNOS or activation of NO_x_ alone does not cause substantial damage to microglia, but the simultaneous production of superoxide and NO by NO_x_ and iNOS has the potential to harm microglia [85,86]. In activated microglia that generate superoxide upon NO_x_ activation, the oxygen and H_2_O_2_ levels quickly become imbalanced and may affect microglial functions. ROS facilitates phagocytosis by amoeboid microglial cells and enhances vesicle formation, which was observed upon treatment of microglial cells with H_2_O_2_ [87]. Microglia-derived ROS can damage adjacent brain cells. Therefore, microglial proliferation and ROS production are potential therapeutic targets that may protect the brain from oxidative damage and neurodegenerative disease [88].

### 4.3. Microglia-Mediated Antioxidant Defense

To prevent oxidative stress by ROS, microglia contain a high cellular GSH concentration and express and upregulate diverse antioxidant enzymes, including SOD, GPx, GR, and catalase. Brain cell cultures labeled with fluorescence showed that microglia express a higher level of GSH than the other cell types in the rat brain [89]. This high concentration of intracellular GSH in microglia contributes to its antioxidant defense system against radical- and peroxide-mediated damage. Microglial cultures stimulated with TNFα showed twice as much GSH as unstimulated microglial cultures [90]. However, the cellular GSH content was lower in microglia treated with LPS/IFNγ, which induce iNOS production, but the mitochondrial GSH content was unaffected [91]. Thus, the microglial GSH content shows a binary effect, in which it increases upon improvements in GSH synthesis and decreases upon accelerated GSH consumption, depending on the type of stimulation. SOD, another antioxidant enzyme, was observed by immunocytochemical staining in activated microglia after quinolinic acid treatment, but was not detected in microglia under basal conditions [92,93]. The specific activity of MnSOD is 20 and 4 times higher in cultured microglia than in cultured astrocytes and oligodendrocytes, respectively [94]. In microglia treated with LPS/IFNγ or TNFα to induce oxidative stress, mitochondrial MnSOD expression was upregulated, which improved the ability of cells to decompose mitochondrial superoxide [90,95]. Elevated SOD activity in activated microglia reduces the risk of cell damage by superoxide-derived hydroxyl radicals and peroxynitrite. The upregulation of GSH peroxidases (GPx) in microglia is also a crucial mechanism against oxidative stress. The specific activity of GPx and GSH reductase (GR) is significantly higher in microglia than in neurons [96,97,98]. However, the specific activity of catalase was similar and/or a little lower in microglia than in other brain cell types, including neurons, astrocytes, and oligodendrocytes [97,99]. Although microglial GSH disulfide (GSSG) increases to almost 30% of total cellular GSH after exposure to H_2_O_2_, microglial GSSG is barely detectable under basal conditions [98,100].

## 5. Neuron–Glia Crosstalk in the Antioxidant Defense Mechanism

Neurons depend on a continuous supply of glucose and oxygen from outside the brain via cerebral blood flow, even though they do not directly contact microvessels. However, 99% of the brain capillary surface is covered with astrocyte end-feet processes, indicating that neurons must interact with astrocytes to receive essential materials from the cerebral circulation [101]. In fact, crosstalk between astrocytes and neurons is essential for neuronal defense against ROS. Activated astrocytes exhibit ambidextrous properties such as A1 and A2 astrocytes. A1 astrocytes lead to neuronal loss by promoting inflammation via the NF-kB pathway, which loses the ability to protect neurons and control synaptogenesis [102,103]. A2 astrocytes promote neuronal survival via the Janus kinase/signal transducer and activator of transcription 3 (JAK-STAT3) signaling pathway by upregulating neurotrophic factors [104].

**Figure 3:**
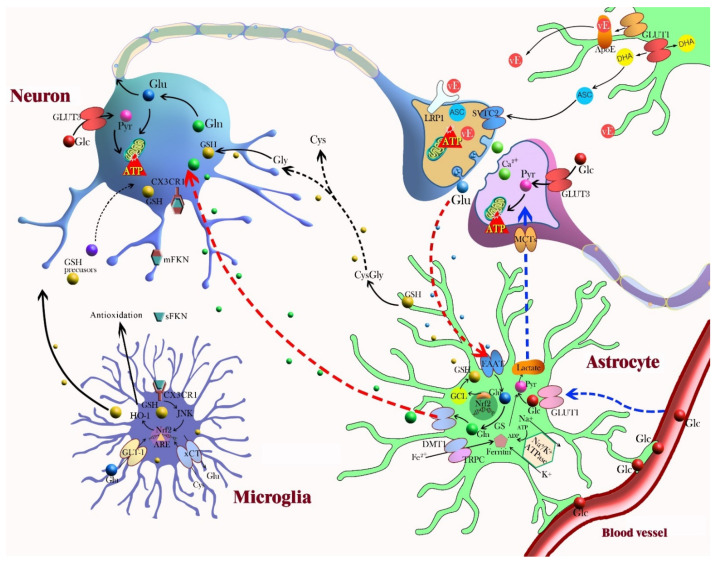
This diagram represents neuron–glia crosstalk involved in neuroprotection and the antioxidant defense mechanism. Astrocyte-neuron: Astrocytes contain a variety of antioxidant molecules, including glutathione (GSH), ascorbate, and vitamin E (vE), and ROS-detoxifying enzymes, such as GSH S-transferase, GSH peroxidase, thioredoxin reductase, and catalase. Astrocytes project the end-feet processes onto brain capillary surface so that astrocytes control the movement of molecules and cells between the vascular compartments and the brain. In the lactate shuttle, astrocytes support neurons by regulating glucose transformation into lactate, which ensures the energetic support of neurons. Neuronal activity triggers glucose metabolism in astrocytes. Glucose is converted to pyruvate by glycolysis and to lactate, which is released from astrocytes and taken up by neurons (blue arrow). Astrocytes can synthesize GSH via activation of Nrf2 and can shuttle GSH precursors to neurons for GSH synthesis. Astrocytes release GSH into the extracellular space and neurons take up the GSH directly or use extracellular neuronal aminopeptidase N to form glycine and cysteine (black arrow). In glutamate uptake and recycling, glutamate from the synaptic space enters astrocytes through EAAT and is converted by glutamine synthetase (GS) to inactive glutamine. After its release and import into neurons, glutamine can be re-converted to glutamate (red arrow). Recycled ascorbate can directly scavenge ROS and act as a cofactor for the recycling of oxidized vE and GSH. Astrocytes take up dehydroascorbic acid (DHA), an oxidation product of ascorbate, from the extracellular space and recycle it back to ascorbic acid. Astrocytes capture and transport excess extracellular K^+^ to the astrocytic syncytium through Na^+^/K^+^ ATPase. Nrf2 induction of glutamate cysteine ligase (GCL) increases GSH synthesis in astrocytes, and GSH is subsequently exported to the extracellular medium. Astrocytes also participate in metal sequestration in the brain to prevent the generation of free radicals by redox active metals. Microglia-neuron: Microglia contain a high cellular GSH concentration and express and upregulate diverse antioxidant enzymes. The expression of classical antioxidant proteins are controlled by Nrf2 in microglia. Heme oxygenase-1 (HO-1), an antioxidant enzyme upregulated by Nrf2, inhibits NOX2 activation. Fractalkine (FKN) is predominantly expressed on neuronal cells, and microglia and neurons exclusively express the fractalkine receptor (CX3CR1); this is an interesting signaling axis for communication. Abbreviations: ARE, antioxidant response element; ASC, ascorbate; ApoE, apolipoprotein E; xCT, cysteine-glutamate exchanger; Cys, cysteine; DHA, dehydroascorbic acid; DMT1, divalent metal transporter; EAAT, excitatory amino acid transporter; mFKN, membrane-anchored fractalkine; sFKN, soluble fractalkine; CX3CR1, fractalkine receptor; Glc, glucose; GLUT, glucose transporter; Glu, glutamate; Gln, glutamine; GSH, glutathione; GCL, glutamate-cysteine ligase; GS, glutamine synthetase; GLAST, glutamate aspartate transporter; GLT1, glutamate transporter 1; Gly, glycine; HO-1, heme oxygenase-1; JNK, c-Jun amino terminal kinase; LRP, lipoprotein receptor-related protein; MCT, monocarboxylate transporter; Nrf2, nuclear erythroid-related factor 2; Pyr, pyruvate; SVTC-2, sodium-dependent transporter; TRPC, transient receptor potential canonical.

Neurons produce glutamate, which stimulates ascorbate release from astrocytes during glutamatergic synaptic activity, and then ascorbate enters neurons and inhibits glucose consumption and stimulates lactate transport. The antioxidant and metabolic interplay between neurons and astrocytes is described in Figure 3. Astrocytes are responsible for the maintenance and support of neurons by regulating oxidative stress via GSH production and glucose transformation into lactate, which ensures the energetic support of neurons [105]. The intrinsic antioxidant GSH, which is produced in both neurons and astrocytes, acts as an independent ROS scavenger and as a substrate for an antioxidant. Neuronal cells depend on astrocyte-derived GSH, for example, neurons depend on shuttling of the GSH precursor from astrocytes to neurons. Cysteine is the rate-limiting substrate for GSH synthesis, and extracellular cysteine is readily auto-oxidized to cystine [53]. Cystine uptake occurs via the cystine/glutamate exchange transporter in astrocytes, and then astrocytes reduce cystine back to cysteine for GSH synthesis. GSH directly reacts with ROS or acts as a substrate for GSH S-transferase or GSH peroxidase [50]. For the efficient use of extracellular cystine as a cysteine precursor, neurons depend on astrocytes to supply cysteine, even though neurons can synthesize GSH [54,106]. It has been shown that neuronal GSH levels are significantly higher when co-cultured with astrocytes [107]. Upon H_2_O_2_-induced oxidative stress, noradrenaline treatment protects neurons by increasing the supply of GSH from astrocytes to neurons via stimulation of the beta3-adenoreceptor in astrocytes [108]. The other interactions between neurons and astrocytes that are related to antioxidant activity include an astrocyte–neuron lactate shuttle and the recycling of ascorbate [55]. Astrocytes play a crucial role in coupling neuronal activity and brain glucose uptake through an astrocyte–neuron lactate shuttle [109]. Neuronal activity triggers glucose metabolism in astrocytes; glucose is converted to pyruvate by glycolysis and converted to lactate, which is released from astrocytes and taken up by neurons for oxidative phosphorylation. Ascorbate that is concentrated in the brain is released from glial reservoirs into the extracellular space and taken up by neurons. Highly activated neurons generate ROS, which oxidize ascorbate to dehydroascorbic acid (DHA), and scavenge ROS by taking up ascorbate [110,111].

In neurotransmitters, overstimulation with glutamate induces excitotoxicity, which is involved in the pathogenesis of many brain disorders. Astrocytes use two main transporters, excitatory amino acid transporter1 (EAAT1)/glutamate aspartate transporter (GLAST) and EAAT2/glutamate transporter-1 (GLT1), to take up glutamate and return glutamate to neurons via the well-established glutamate–glutamine cycle that involves the astrocyte-specific enzyme glutamine synthetase (GS), which converts glutamine into glutamate. If failure to convert glutamine back to glutamate occurs, the glutamate pool in presynaptic terminals would rapidly be depleted and excitatory neurotransmission would be disrupted [112,113]. An insufficient supply of glutamine to GABAergic neurons induces GABAergic dysfunction [114,115]. Glutamine in astrocytes is critical for GABA replenishment by glutamate decarboxylase, known as the GABA–glutamine cycle, in GABAergic neurons [116]. Neuronal activity and action potentials increase extracellular K^+^ in restricted spaces and lead to hyper-excitable membrane potentials when tight regulatory mechanisms are absent [117]. Astrocytes have a high number of membrane K^+^ channels and high K^+^ permeability [118,119]. Astrocytes capture and transport excess extracellular K^+^ to the astrocytic syncytium through Na^+^/K^+^ ATPase. Astrocytes also regulate the Ca^2+^ concentration within neurons via astrocytic calcium signaling and astrocyte-neuron crosstalk. Neuronal activation, which induces a reduction in extracellular Ca^2+^, evokes spatiotemporal changes via the Ca^2+^/Na^+^ exchanger in astrocytes and generates astrocytic Ca^2+^ waves that propagate from the cytoplasm into the extracellular space [120,121]. Astrocytes are also highly mechanosensitive, and a drop in extracellular Ca^2+^ due to synaptic activity leads to the release of ATP from astrocytes via the opening of connexin 43 hemichannels [122,123,124]. Neuronal activity can elicit metabolic changes in astrocytes via dual Na^+^ and Ca^2+^ signaling, which triggers glucose mobilization and glycolysis to support neuronal function. Astrocytic metabolism correlates with the high metabolic demands from neurons [125,126].

The differential antioxidant response of neurons and astrocytes results from the preferential astrocytic expression of Nrf2, a redox-sensitive transcription factor. Nrf2-ARE is a critical pathway for the regulation of the antioxidant defense mechanism because it regulates the expression of phase II detoxifying enzymes and antioxidant genes [127]. The higher susceptibility of neurons to ROS is due to the continuous destabilization and degradation of the antioxidant transcriptional activator Nrf2, which regulates the GSH system, the thioredoxin system, and SOD [128,129]. Nrf2 is more stable in astrocytes; thus, they dispose of the ROS in the nervous system. Nrf2 induction of glutamate cysteine ligase (GCL) increases GSH synthesis in astrocytes, and GSH precursors are subsequently exported to the extracellular medium [130]. Moreover, Nrf2-induced GSH synthesis in astrocytes is used to replenish neuronal GSH through the astrocyte-neuron shuttle. Nrf2-induced molecules, such as GSH-related enzymes and metallothioneins, are more highly expressed in astrocytes than in neurons, indicating that Nrf2 activation in astrocytes protects neurons from oxidative stress [131,132].

Microglia exhibit a surveying phenotype via dynamic crosstalk between microglia and neurons in the healthy brain [133]. M1 microglia promote inflammation by producing proinflammatory cytokines and inducing NO synthase activity. M2 microglia regulate immune function and promote repair by secreting anti-inflammatory cytokines [134,135]. The function of redox regulators in microglia is unclear, but many antioxidant proteins are linked to inflammation via functional microglia. In the crosstalk between microglia and neurons described in Figure 3, the expression of classical antioxidant proteins is controlled by Nrf2 in microglia [6]. Nrf2 deficiency exacerbates cognitive impairment and reactive microgliosis upon LPS treatment in vivo [136]. Heme oxygenase-1 (HO-1), an antioxidant enzyme upregulated by Nrf2, inhibits NOX2 activation upon stimulation with LPS [137]. HO-1, which may facilitate the attenuation of TLR4 signaling by NOX inhibition, is responsible for the conversion of heme to biliverdin and carbon monoxide and functions as an antioxidant enzyme [138]. The overexpression of HO-1 in microglia reduced neurotoxic iron accumulation in aged mice [139]. The genetic deletion of microglial-specific proteins and mechanistic interruption of neuronal activity by microglia manipulation showed that microglia modulate neuronal activity. Fractalkine (FKN) is predominantly expressed in the CNS and localized on neuronal cells. The FKN receptor (CX3CR1) is exclusively expressed on microglia and neurons and is an interesting signaling axis for communication between microglia and neurons [69,140]. A CX3CR1 deficiency was linked to the disruption of neurogenesis and neural connectivity [141]. DAP12 is another microglia-specific protein which occurs as a result of alterations in glutamate receptor content at synapse through microglial BDNF [142]. In neurotransmission with microglia-specific manipulation, microglia-conditioned media enhanced excitatory postsynaptic potentials and current in dissociated cell cultures [143]. The inhibition of microglial activation by minocycline reduced neuronal cell death and spontaneous recurrent seizures in a rat lithium–pilocarpine model [144].

## 6. Conclusions

Neurons, which have high energy demands, engage in metabolic and redox crosstalk with surrounding cells for normal brain function. Glia play essential roles in the redox and metabolic needs of neurons for neurotransmission and survival. Several previous studies have demonstrated the molecular and cellular aspects of this glia–neuronal coupling and have used antioxidant therapies to slow down the progression of neurodegeneration [139,145,146,147]. We reviewed oxidant and antioxidant systems in activated due to paracrine redox signaling and the crucial role of neuron–glia crosstalk against oxidative stress in the CNS, where the extracellular space and distance to neighboring cells or cell structures is extremely limited. Glial cells show morphological and molecular alterations in response to oxidative injury and regulate neuronal activities under these conditions. This neuron–glia communication plays a critical role in oxidative conditions by delaying neurodegeneration and aberrant neurogenesis via redox-balancing mechanisms.

## Data Availability

None.

## Abbreviations

AD	Alzheimer’s disease
ApoE	apolipoprotein E
ARE	antioxidant response element
ASC	ascorbate
ATP	adenosine triphosphate
CNS	central nervous system
CX3CR1	fractalkine receptor
Cys	cysteine
DAP12	DNAX activation protein of 12 kDa
DHA	dehydroascorbic acid
DMT1	divalent metal transporter
DRD2	dopamine d2 receptor
EAAT	excitatory amino acid transporter
FKN	fractalkine
mFKN	membrane-anchored fractalkine
sFKN	soluble fractalkine
GCL	glutamate cysteine ligase
GDNF	glial cell-line-derived neurotrophic factor
GLAST	glutamate aspartate transporter
Glc	glucose
Gln	glutamine
GLT	glutamate transporter
Glu	glutamate
GLUT	glucose transporter
Gly	glycine
GPx	GSH peroxidases
GR	GSH reductase
GS	glutamine synthetase
GSH	glutathione
GSSG	GSH disulfide
HO-1	heme oxygenase-1
IFNγ	interferon-γ
JAK-STAT3	janus kinase/signal transducer and activator of transcription 3
Keap1	kelch-like ech-associated protein
LRP	lipoprotein receptor-related protein
LPS	lipopolysaccharide
MCT	monocarboxylate transporter
NADPH	nicotinamide adenine dinucleotide phosphate
NGF	nerve growth factor
NLR	leucine-rich-repeat
NLRP3	leucine-rich-repeat pyrin domain-containing 3
NO	nitric oxide
NOS	nitric oxide synthase
NO_x_	nitrates/nitrites
NOX	NADPH oxidase
NQO1	quinone oxidoreductase 1
Nrf2	nuclear erythroid-related factor 2
O_2_	oxygen
PD	parkinson’s disease
Pry	pyruvate
RNS	reactive nitrogen species
ROS	reactive oxygen species
SOD	superoxide dismutase
SVTC-2	sodium-dependent transporter
tBHQ	tertiary butylhydroquinone
TLRs	toll-like receptors
TNF	tumor necrosis factor
TRPC	transient receptor potential canonical
vE	vitamin E
xCT	cysteine–glutamate exchanger
